# Antiproliferative activity of PEP005, a novel ingenol angelate that modulates PKC functions, alone and in combination with cytotoxic agents in human colon cancer cells

**DOI:** 10.1038/sj.bjc.6604642

**Published:** 2008-11-25

**Authors:** K A Benhadji, M Serova, A Ghoul, E Cvitkovic, C Le Tourneau, S M Ogbourne, F Lokiec, F Calvo, P Hammel, S Faivre, E Raymond

**Affiliations:** 1 INSERM U728, RayLab, Department of Medical Oncology, Beaujon University Hospital, APHP, Paris 7, 100 boulevard Général Leclerc, Clichy 92110, France; 2 Department of clinical Pharmacology, René Huguenin Cancer Center, 35 rue Dailly, Saint-Cloud 92210, France; 3 Peplin Ltd, 1 Breakfast Creek Road, Newstead Brisbane, Queensland 4006, Australia; 4 INSERM U716 IGM-Saint-Louis Hospital, APHP, Paris 7, 27 rue Juliette Dodu, Paris 75010, France

**Keywords:** PKC*α*, PKC*δ*, P38, drug resistance, schedule dependency

## Abstract

PEP005 is a novel ingenol angelate that modulates protein kinases C (PKC) functions by activating PKC*δ* and inhibiting PKC*α*. This study assessed the antiproliferative effects of PEP005 alone and in combination with several other anticancer agents in a panel of 10 human cancer cell lines characterised for expression of several PKC isoforms. PEP005 displayed antiproliferative effects at clinically relevant concentrations with a unique cytotoxicity profile that differs from that of most other investigated cytotoxic agents, including staurosporine. In a subset of colon cancer cells, the IC_50_ of PEP005 ranged from 0.01–140 *μ*M. The antiproliferative effects of PEP005 were shown to be concentration- and time-dependent. In Colo205 cells, apoptosis induction was observed at concentrations ranging from 0.03 to 3 *μ*M. Exposure to PEP005 also induced accumulation of cells in the G1 phase of the cell cycle. In addition, PEP005 increased the phosphorylation of PKC*δ* and p38. In Colo205 cells, combinations of PEP005 with several cytotoxic agents including oxaliplatin, SN38, 5FU, gemcitabine, doxorubicin, vinorelbine, and docetaxel yielded sequence-dependent antiproliferative effects. Cell cycle blockage induced by PEP005 in late G1 lasted for up to 24 h and therefore a 24 h lag-time between PEP005 and subsequent exposure to cytotoxics was required to optimise PEP005 combinations with several anticancer agents. These data support further evaluation of PEP005 as an anticancer agent and may help to optimise clinical trials with PEP005-based combinations in patients with solid tumours.

Protein kinase C (PKC) is a family of serine/threonine kinases comprising at least 12 isoenzymes that are effectors of diacylglycerol and the main targets of phorbol-ester tumour promoters (Ghoul *et al*, 2005). Protein kinases C have been shown to play an important role in a variety of cellular events, including cellular proliferation, cell cycle progression, differentiation, drug efflux, apoptosis, and tumour angiogenesis (Serova *et al*, 2006). Evidence has suggested that selective targeting of PKC may enhance the therapeutic efficacy of established chemotherapeutic agents and sensitise cancer cells to ionising radiation (Mackay and Twelves, 2007). Various PKC modulators with promising anticancer activity as single agents have been tested to reverse resistance to cytotoxic chemotherapy, providing a rationale for the development of drug combinations with PKC modulators in a number of human tumours.

The expression of PKC isozymes in tumours has been shown to be partially tissue specific (Pongracz *et al*, 1995; Verstovsek *et al*, 1998; Griner and Kazanietz, 2007). Protein kinase C*α* has been shown to play a role in cellular proliferation and invasion, PKC*β* seems to promote invasion (Zhang *et al*, 2004), the *β*II isoform being an important mediator of VEGF-induced angiogenesis (Teicher *et al*, 2001; Suzuma *et al*, 2002), and PKC*ε* was shown to promote cell survival through the activation of the AKT pathway and upregulation of several survival factors. In addition, PKC*δ* was shown to play a key role in cell survival and apoptosis. Protein kinase C*δ* may be activated in response to Ara-C as demonstrated in U937 human leukaemia cells (Emoto *et al*, 1996), cisplatin in H69 human small-cell lung cancer cells (Persaud *et al*, 2005), and etoposide in human glioma cells (Blass *et al*, 2002). Interestingly, apoptosis was severely impaired when the PKC*δ* function or expression was inhibited (Humphries *et al*, 2006). In addition, activation of PKC*δ* may cause G1 and G2 cell cycle arrest through the modulation of cyclin and cyclin-dependent kinase expression (Griner and Kazanietz, 2007). Thus, PKC*δ* is regarded as an attractive target for therapeutic interventions.

PEP005 is a novel ingenol angelate extracted and purified from *Euphorbia peplus*. Chemically, PEP005 is structurally analogous to phorbol esters (Figure 1) and is a potent modulator of PKC isoenzymes (Kedei *et al*, 2004). PEP005 is currently being developed as a topical treatment for actinic keratoses and basal cell carcinoma (www.cancertrials.gov). In earlier studies, PEP005 was shown to modulate PKCs by activating PKC*δ* in human myeloid leukaemia cancer cell lines, thereby inducing cellular apoptosis (Hampson *et al*, 2005) in melanoma models, inducing senescence through PKC-mediated activation of the MAPK pathway (Cozzi *et al*, 2006) and in colon cancer models, inducing apoptosis through the inhibition of the AKT signalling pathway (Serova *et al*., 2008). PEP005 appears to be selective for growth inhibition of cancer cells as it does not inhibit the growth of human neonatal fibroblasts (Cozzi *et al*, 2006) and does not induce apoptosis in normal CD34+ cord blood myeloblasts (Hampson *et al*, 2005). In leukaemia cells, resistance to PEP005 was associated with the failure to express PKC*δ*. In solid tumours, PEP005 was shown to induce necrosis in human melanoma, cervical cancer, and prostate xenografts (Ogbourne *et al*, 2004).

The aim of our study was to investigate antiproliferative effects of PEP005 in a panel of colon cancer cell lines that expressed various levels of PKC*δ* and to evaluate the effects of PEP005-based combinations with cytotoxic agents commonly used for the treatment of cancer.

## Materials and methods

### Cell lines and reagents

The HT29 colon cancer, MCF7 breast cancer, and OVCAR3 ovarian cancer cell lines were obtained from the American Type Culture Collection (Rockville, MD, USA) and maintained according to its recommendations. Colo205, HCT116, HCC2998 colon, HOP62 and HOP92 lung, IGROV1 ovarian, and MDA-MB-435 breast cancer cell lines were obtained from NCI (Bethesda, MD, USA). All cell lines were regularly tested for mycoplasma contamination by PCR using a Stratagene kit (Austin, TX, USA).

Purified PEP005 was supplied by Peplin Ltd (Brisbane, Australia). Cytotoxic agents were prepared in DMSO or other solvents, as appropriate. Cisplatin (Sigma, St Quentin Fallavier, France), oxaliplatin (Sanofi-Aventis, Paris, France), doxorubicin (Teva, Paris, France), gemcitabine (Lilly, Suresnes, France), 5FU (Teva, France), vinorelbine (Pierre Fabre, Boulogne, France), docetaxel (Sanofi-Aventis, France), and staurosporine (Sigma, France) were used.

### *In vitro* growth inhibition assay (MTT assay)

The data generated were from three separate experiments, each performed in duplicate. Cell viability was determined using the MTT assay, which was carried out as described previously (Hansen *et al*, 1989). Briefly, cells were seeded in 96-well plates at a density of 2 × 10^3^ cells per well. For single-agent studies, cells were seeded and allowed to settle for 24 h before treatment with increasing concentrations of PEP005 or cytotoxics. After incubation, the cells were allowed to recover in compound-free medium for 72 h, before determination of growth inhibition using the MTT assay. Cells were incubated with 0.4 mg ml^−1^ of MTT dye (3-[4, 5-dimethylthiazol-2-yl]-2, 5-diphenyltetrazolium bromide; Sigma, France) for 4 h at 37°C. The monolayer was suspended in 0.1 ml of DMSO and the absorbance at 560 nm was measured using a microplate reader (Thermo, Villebon-sur-Yvette, France). The control value corresponding to untreated cells was taken as 100% and the viability of treated samples was expressed as a percentage of the control. The IC_50_ values were determined as the concentration that reduced cell viability by 50%.

### Combination studies

For simultaneous drug exposure, cells were seeded at 2 × 10^3^ cells per well in 96-well plates and treated 24 h later with increasing concentrations of PEP005 alone or with concentrations of cytotoxic agents corresponding to the IC_20_, IC_40_, IC_60_, or IC_80_ values. Drugs were either added simultaneously for 24 h or given using sequential exposures. For sequential exposures, cells were incubated with different concentrations of PEP005 for 24 h before or following treatment with other drugs. The cells were washed twice in drug-free medium then post-incubated in drug-free medium for 72 h. Growth inhibition was then determined by the MTT assay. A washout period of 24 h between exposures to the two drugs, followed by 48 h in drug-free culture medium was also studied.

### Statistical analysis and determination of synergistic activity

Drug combination effects were determined using the Chou and Talalay method as described elsewhere (Chou and Talalay, 1984) based on the median effect principle. The combination index (CI) values of <1 indicate synergy, a value of 1 indicates additive effects, and a value of >1 indicates antagonism. Variability between experiments led us to consider that CI values ranging from 0.8 to 1.2 represent additive effects. Thus a CI below 0.8 indicates synergy, above 1.2 antagonism, and between 0.8 and 1.2 additive effects. Data were analysed using concentration effect analysis software (Biosoft, Cambridge, UK). For statistical analysis and graphs, Prism software (GraphPad, CA, USA) was used. Experiments were performed three times, in duplicate. Means and standard deviations were compared using Student's *t*-test (two-sided *P*-value).

### Cell cycle analysis

Cell cycle analysis were studied by flow cytometry. In brief, cells were seeded in 25 cm^3^ flasks and treated with various concentrations of PEP005 or cytotoxics. At various time points, adherent and non-adherent cells were recovered, washed twice in PBS, fixed in 70% ethanol, and stored at 4°C until analysed. Cells were washed twice in PBS, incubated for 20 min at room temperature with 250 *μ*g ml^−1^ RNAse A and 20 min at 4°C with 50 *μ*g ml^−1^ propidium iodide (PI). The cell cycle distribution and percentage of apoptotic cells were determined using a FACScan flow cytometer (Becton Dickinson, Le-Pont-de-Claix, NJ, USA).

### Apoptosis

The measurement of the percentage of apoptotic cells was assessed by flow cytometry using double staining annexin V-FITC and PI. The Annexin V kit (Sigma, France) was used. Cells were prepared according to the manufacturer's protocol. Cell distribution was determined using a FACScan flow cytometer (Becton Dickinson).

### Western blot analysis

Cells were lysed in buffer containing 50 mM HEPES (pH 7.6), 150 mM NaCl, 1% Triton X-100, 2 mM vanadate, 100 mM NaF, and 0.40 mg ml^−1^ phenylmethyl fluoride. Equal amounts of protein (20 *μ*g per lane) were subjected to SDS–PAGE and transferred to nitrocellulose membranes. Membranes were blocked with 5% milk in 0.05% Tween-20/TBS and then incubated with the first antibody overnight. Membranes were then incubated with the secondary anti-mouse or anti-rabbit horseradish peroxidase conjugated antibody. Bands were visualised using the enhanced chemiluminescence Western blotting detection system (ECL, Amersham, NJ, USA). Densitometric analysis was performed under conditions that yielded a linear response. The following antibodies were used: anti-PKCs *α*, *β*, *δ*, and *ε* (Cell Signalling, Saint Quentin Yvelines, France), anti-phospho-PKC *δ* (Upstate Biotechnology, Hampshire, UK), and anti-phospho-p38 (BD Biosciences, Le-Pont-de-Claix, France).

## Results

### Antiproliferative effects of PEP005 in cancer cell lines with various PKC expressions

**Figure 2:**
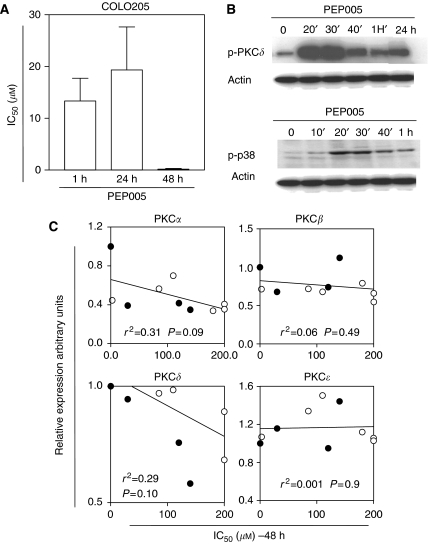
Antiproliferative effects of PEP005 in cells, according to PKC expression. (**A**) Cytotoxicity of PEP005 after 1, 24, and 48 h exposures in Colo205 cells. (**B**) Modulation of PKC *δ* and p38 phosphorylation by PEP005 in Colo205 cells. Colo205 cells were treated with PEP005 (0.3 *μ*M) for the indicated time. Protein extracts were then analysed for PKC *δ* and p38 phosphorylation. (**C**) Correlation between PKC levels and sensitivity to PEP005. Expression levels of PKC *α*, *β*, *δ*, and *ε* were correlated with IC_50_ of PEP005 in 10 cancer cell lines (•, colon cancer cell lines; ○, other cell lines). All values from the densitometric analysis of western blot are standardised with respect to Colo205. The *r*^2^ value is the correlation coefficient calculated by linear regression analysis.

We first evaluated the expression of PKC isoforms *α*, *β*, *δ*, and *ε* in a panel of 10 human cancer cell lines representing common human cancers (breast, lung, colon, and ovarian cancer). The mRNA and protein expression levels of PKCs were assessed by RT–PCR (data not shown) and western blot analysis. Protein kinase C isoforms *α* and *δ*, which are targets of PEP005, were expressed at various levels in the panel of cell lines. On the basis of these results, we selected four colon cancer cell lines for further experiments. Those cells were considered as representative as they displayed different levels of PKC*α* and PKC*δ* expression. The antiproliferative effects were assessed by MTT assay for durations of exposure varying between 1 and 48 h (Table 1). In our panel of colon cancer cells, exposure to PEP005 for 48 h led to higher antiproliferative effects than shorter exposure times (Table 1 and Figure 2A). Colo205, HCC2998, HCT116, and HT29 cells displayed IC_50_ values of 0.01, 30.0, 120.0, and 140.0 *μ*M after 48 h PEP005 exposure, respectively. Previous data showed no relevant change in the expression of PKC isoforms under treatment with PEP005 in cancer cells but that PEP005 may activate PKC and inhibit PKC phosphorylation (Serova *et al*, 2008). As expected from our previous results, we observed that exposure of Colo205 cells to 3 *μ*M PEP005 for 20 min increased the phosphorylation of PKC*δ* and activated p38 (Figure 2B). Semiquantitative western blot analysis of expression of PKC isoforms *α*, *β*, *δ*, and *ε* was performed showing that most cancer cells in our panel expressed various levels of PKC. Attempts were made to determine whether baseline expressions of those PKC isoforms correlated with sensitivity to PEP005. In this study, we further found no statistically significant correlation between sensitivity to PEP005, and PKC isoforms expression was detectable neither in our panel of colon cancer cell lines nor in the extended panel of 10 human cancer cell lines (Figure 2C). The cytotoxicity profile of PEP005 was compared to that of various chemotherapeutic agents including oxaliplatin, cisplatin, doxorubicin, SN38, 5FU, gemcitabine, vinorelbine, docetaxel, and staurosporine in our panel of colon cancer cell lines. The IC_50_ values are reported in Table 1. In our study, PEP005 displayed a cytotoxicity profile that differs from that of most cytotoxic agents. Interestingly, Colo205 cells were more sensitive to PEP005 than to 5FU and oxaliplatin.

### Effects of PEP005 on cell cycle and apoptosis

**Figure 3:**
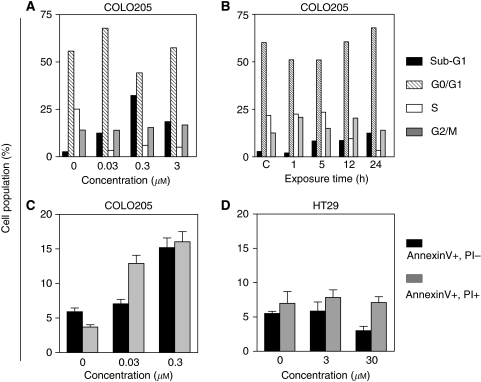
Cell cycle effects and apoptosis induction by PEP005 in colon cancer cells. Effects of increasing concentrations of PEP005 for 24 h (**A**) on cell cycle distribution in Colo205 cells detected by flow cytometry, SubG1, G0/G1, S, and G2/M phases of cell cycle. Effect of duration of exposure to PEP005 (**B**) in Colo205 cells were harvested after different incubation times with 0.3 *μ*M PEP005. (**C**, **D**) Flow cytometry study of PEP005 induced apoptosis in Colo205 and HT29 cell lines. Apoptotic cells are distributed as: early apoptosis (Annexin V positive and PI negative) and late apoptosis (Annexin V and PI positive).

In Colo205 cells, which were the most PEP005 sensitive cells in our panel, we further analysed the cell cycle distribution using flow cytometry. Cells were incubated for 24 h with various concentrations of PEP005. Exposure to PEP005 concentrations ranging from 0.03 to 3.0 *μ*M led to an inhibition of S-phase entry with an accumulation of cells in G1 and an increase of the sub-G1 fraction suggesting apoptosis (Figure 3A). Duration of exposure was shown to play a role in PEP005-induced effects on the cell cycle, the maximal inhibition being obtained for 24 h exposure (Figure 3B). In contrast to that observed in Colo205 cells, no change was observed in HT29 cells that appeared to be more resistant than Colo205 cells to PEP005. As the increase of the sub-G1 fraction suggested induction of apoptosis, we further confirmed apoptosis induction using double staining with annexin V-FITC and PI in cells exposed for 24 h to PEP005 at concentrations ranging 0.03–0.3 *μ*M (Figure 3C). In Colo205 cells, we observed a significant increase of the early apoptosis fraction (annexin V positive, PI negative) as well as of the late apoptosis fraction (annexin V positive, PI positive). In contrast, no apoptosis was detectable in HT29 cells at concentrations ranging from 3 to 30 *μ*M (Figure 3D).

### Antiproliferative effects of PEP005-based combinations

Four schedules were used to study the effects of sequential and simultaneous exposures of PEP005-based combinations. Combinations were made with the cytotoxic agents most frequently used for the treatment of colorectal cancer: oxaliplatin, SN38 (the active metabolite of irinotecan) and 5FU as well as several other anticancer drugs. In schedule 1, PEP005 was added simultaneously with other drugs for 24 h (except for 5FU that was given for 48 h). Schedule 2 used a sequential administration of PEP005 for 24 h before other drugs. Schedule 3 consisted of the opposite sequence giving cytotoxics before PEP005. Schedule 4 gave PEP005 first followed by a 24 h washout period before administration of the second drug. Following incubation, effects were determined using CIs as described previously (Chou and Talalay, 1984). Results of PEP005-combination studies are summarised in Table 2.

**Figure 4:**
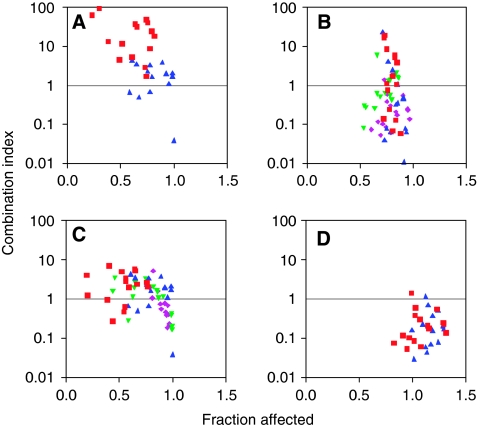
PEP005–oxaliplatin combinations in Colo205 colon cancer cells. The following four schedules were investigated using the Chou and Talalay method: (**A**) Exposure of PEP005 for 24 h along with oxaliplatin; (**B**) PEP005 exposure for 24 h followed by 24 h exposure to oxaliplatin; (**C**) 24 h exposure to oxaliplatin followed by 24 h exposure to PEP005; and (**D**) 24 h exposure to PEP005 followed by a 24 h washout period then followed by exposure to oxaliplatin for 24 h.

Antagonistic effects between PEP005 and cisplatin were observed for all schedules of administration. Concomitant or sequential exposure to PEP005 and oxaliplatin appeared antagonistic in Colo205 cells. However, synergistic effects between the two drugs were observed when a 24 h washout period was included (schedule 4). Synergism between oxaliplatin and PEP005 was observed when Colo205 cells were exposed to oxaliplatin 24 h and 72 h after PEP005 (Figure 4). Combinations of PEP005 with SN38 were also synergistic using sequential exposure. Combinations of PEP005 with 5FU led to various degrees of additive or synergistic activity regardless of the sequence of drug exposure except for concomitant treatment.

We also studied combinations of PEP005 with several other anticancer agents. Synergistic effects were observed with doxorubicin for schedules 2 and 3. The sequence of gemcitabine followed by PEP005 was additive contrasting with the antagonism seen when drugs were administered concomitantly or when PEP005 was given before gemcitabine. This antagonism was reversed when gemcitabine was administered 24 h after PEP005 (schedule 4), the combination being synergistic. In addition, this study showed that sequential exposure to tubulin inhibitors including vinorelbine and docetaxel with PEP005 were additive.

**Figure 5:**
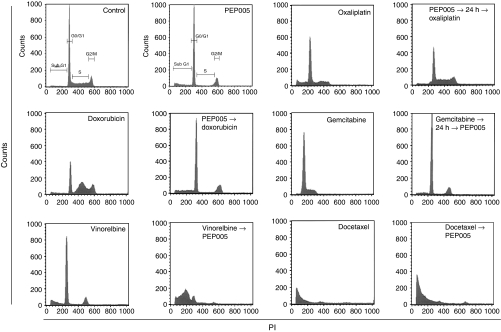
Cell cycle effects of anticancer agents alone and in combination with PEP005. Cells were incubated with the indicated drug sequence at IC_50_ concentrations, washed, and analysed by flow cytometry at 24 h post-incubation in drug-free medium.

Colo205 cells treated with PEP005-based synergistic combinations were analysed using flow cytometry. Cell cycle distribution, displayed in Figure 5, showed sub-G1 accumulation suggesting apoptosis as a result of PEP005 combinations with doxorubicin, gemcitabine, docetaxel, and vinorelbine. Exposure to oxaliplatin 24 h after the end of exposure to PEP005 led to accumulation of cells in S-phase of cell cycle.

## Discussion

Protein kinases C have emerged as novel therapeutic targets for cancer treatment. A large variety of structurally and mechanistically distinct PKC modulators have been identified and/or developed as anticancer agents. Protein kinase C modulators such as bryostatin-1, UCN-01, PKC-412, and PKC*α* antisense oligonucleotide (aprinocarsen) have been extensively tested in clinical trials. Although these agents displayed promising results in *in vitro* and *in vivo* experiments, clinical results remained so far disappointing (Mackay and Twelves, 2007). Only one compound, aprinocarsen, reached phase III and has failed to demonstrate benefit in nonsmall-cell lung carcinoma (Paz-Ares *et al*, 2006). Researches of novel PKC inhibitors are still ongoing, and new compounds such as enzastaurin displayed promising preclinical and clinical activity and are currently being evaluated in phase III trials (Ma and Rosen, 2007). Discrepancies between preclinical research and results of clinical trials with PKC modulators pointed out the need to develop more selective PKC modulators along with identifying predictive molecular markers that may allow selecting patients. In addition, several studies have shown that combinations of PKC modulators with conventional cytotoxics may need preclinical evaluation to determine optimal sequences of administration before entering clinical trials. Studies with bryostatin-1 have emphasised the importance of the schedule sequence, the administration of bryostatin-1 before cisplatin, vincristine and gemcitabine being synergistic, while synergy with paclitaxel required the administration of bryostatin-1 after paclitaxel (Koutcher *et al*, 2000).

Among new compounds, PEP005 appears to be a promising agent for anticancer treatment. In previously published studies, PEP005 displayed antiproliferative effects in several human cancer cell lines (Gillespie *et al*, 2004; Ogbourne *et al*, 2004; Hampson *et al*, 2005; Cozzi *et al*, 2006). Studies that evaluated the activity of PEP005 as a topical agent for human and mouse xenografts have shown that the predominant mechanism of cell death is necrosis (Ogbourne *et al*, 2004) and in some melanoma cell lines apoptosis was observed (Gillespie *et al*, 2004). The capacity of PEP005 to induce apoptosis was further confirmed using leukaemia cancer cells (Hampson *et al*, 2005). In addition, PEP005 induced cell senescence (Cozzi *et al*, 2006). We have recently tested the antiproliferative effects of PEP005 in several human cancer cell lines and we have shown that PEP005 may induce apoptosis in colon cancer cell lines at clinically relevant concentrations (Serova *et al*, 2008). We found that the antiproliferative effects were related to cell cycle inhibition in the G1 phase as well as the induction of apoptosis. These effects may be obtained with a wide range of clinically relevant concentrations. As suggested by our results, antiproliferative effects may be further optimised by increasing the duration of exposure to PEP005 to 48 h. These data warrant pursuing PEP005 clinical development in solid tumours.

Comparison of the cytotoxic effects of PEP005 with those of other anticancer agents confirmed its unique cytotoxic profile, which is related to its specific mechanism of action. In this study, we found no correlation between PKC isoforms expression and the antiproliferative effects of PEP005. We previously showed that PEP005 induced activation of PKC*δ* and reduced expression of PKC*α* resulting in apoptosis mediated by activation of Ras/Raf/MAPK and inhibition of PI3K/AKT pathways in Colo205 cells (Serova *et al*, 2008). In contrast, in the HT29 resistant cell line, no activation of PKC*δ*, MAPK, or AKT was detectable. Further studies may identify molecular markers to help predict sensitivity and/or resistance to PEP005 in human solid tumours.

Considering that concentrations required to observe cytotoxic effects, apoptosis, and/or cell cycle blockage may be limited by toxicity when PEP005 is given to patients with solid tumours, we considered that it is essential to evaluate combinations which could allow the use of lower concentrations of PEP005 and which might improve the cytotoxic effects of already used anticancer agents. In this study, we showed that PEP005 has additive and/or synergistic effects when combined with a large variety of classical cytotoxic drugs such as oxaliplatin, cisplatin, doxorubicin, SN38, 5FU, gemcitabine, vinorelbine, and docetaxel. However, strong evidences of schedule dependency were observed. For example, concomitant administrations of PEP005 and several cytotoxics were mainly antagonistic, suggesting that the cell cycle arrest induced by PEP005 may prevent the cytotoxicity of drugs that target DNA and microtubules. Interestingly, considering that cell cycle blockage induced by PEP005 in late G1 may last for up to 24 h, we further considered experiments introducing a 24 h lag time between PEP005 and subsequent exposure to cytotoxics. This finding was observed with oxaliplatin, a major drug used for the treatment of colon cancer. Known mechanisms of action of oxaliplatin may partly explain our findings. For instance, oxaliplatin cytotoxicity is known to be related to platinum-DNA adduct formation resulting in cell cycle blockage in the G2/M phase of cell cycle. Cell cycle arrest by PEP005 in phase G1 may result in decreasing the window of opportunity for incorporation of platinum into DNA and also increasing duration of DNA repair during G1. Further studies looking at platinum-DNA adducts formation may further provide insight on mechanisms resulting into antagonistic effects. However, introducing a 24 h lag time between PEP005 and oxaliplatin may allow cells to re-enter cell cycle, thus providing more opportunity for platinum-DNA adduct formation and reducing the duration during which repair of platinum-DNA adducts occur, thereby increasing the cytotoxicity of the combination. In contrast, PEP005 combinations with 5FU were shown to be synergistic, regardless of schedules, which might be, at least in part, explained by known effects of 5FU on cell cycle that does depend on duration of the G0/G1 phase of cell cycle.

While further *in vivo* studies are warranted to confirm the effectiveness of PEP005 combinations with cytotoxic agents, our finding may help designing trials in which a lag time between PEP005 and cytotoxic may be introduced to optimise cytotoxic activity.

In summary, modulation of PKC by simultaneous activation of PKC*δ* and inhibition of PKC*α* using PEP005, displayed antiproliferative effects due to late G1 cell cycle arrest and/or apoptosis induction at concentrations that may be reached in clinical trials. These effects appear time dependent and are likely to require prolonged exposure to PEP005. Combinations with other cytotoxic drugs may require giving administration of PEP005 before cytotoxics with a lag time of 24 h. Thus far, no target marker has been identified that may predict PEP005 activity in colon cancer cell lines, thus warranting further preclinical research aimed toward detecting molecular surrogate markers of sensitivity to PEP005.

## Figures and Tables

**Figure 1:**
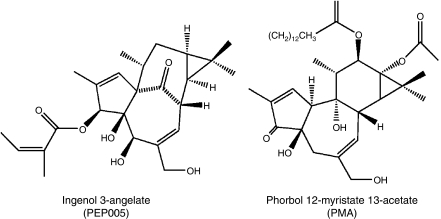
Chemical structure of PEP005 and phorbol 12-myristate 13-acetate (PMA).

**Table 1:** Antiproliferative effects (IC_50_ using MTT assay) of PEP005, staurosporine, and several anticancer agents in a panel of human colon cancer cell lines

	**IC_50_ (*μ*M)**			
	**HT29**	**HCT116**	**HCC2998**	**COLO205**
PEP005 (1 h)	>300	>300	300	35±7
PEP005 (24 h)	>100	>300	>300	3±1.4
PEP005 (48 h)	140±28	120±24	30±6	0.01±0.002
Staurosporine	0.210±0.01	0.013±0.003	0.080±0.016	0.029±0.001
Cisplatine	25.00±3.33	9.15±0.85	25±5	9.5±2.5
Oxaliplatine	60±12	20±4	4±1.41	8±1.6
Doxorubicin	1.07±0.16	0.10±0.02	0.70±0.14	1.5±0.5
Gemcitabine	0.04±0.008	0.05±0.01	0.04±0.008	20±4
5FU	23.50±0.50	5.25±1.25	10±0.2	240±20
Vinorelbine	0.01±0.002	0.01±0	0.015±0.005	0.02±0.004
Docetaxel	0.001±0.0002	0.4± 0.08	0.0008± 0.0001	0.007±0.001

Cells were treated with PEP005 for 1, 24, or 48 h and other drugs for 24 h.

**Table 2:** Mean combination index values for PEP005-based combinations in Colo205 colon cancer cells

**Combinations with**	**Combination index mean (CI 95%)**
*Cisplatin*	
A	1.52 (1.03–2.02)
B	8.85 (2.79–14.91)
C	24.91 (−8.96 to 58.79)
D	1.49 (0.23–2.74)
	
*Oxaliplatin*	
A	18.67 (6.58–30.76)
B	1.95 (0.85–3.0)
C	1.81 (1.41–2.21)
D	0.28 (0.17–0.4)
	
*SN38*	
B	0.53 (0.10–0.97)
C	0.70 (0.48–0.92)
	
*5FU*	
A	1.49 (1.19–1.80)
B	0.71 (0.31–0.80)
C	1.09 (0.52–1.66)
D	0.56 (0.31–0.81)
	
*Doxorubicin*	
A	1.04 (0.73–1.36)
B	0.74 (0.46–1.02)
C	0.92 (0.46–1.38)
D	1.13 (0.8–1.46)
	
*Gemcitabine*	
A	1.77 (1.32–2.22)
B	7.29 (0.58–14.01)
C	1.17 (0.83–1.51)
D	0.68 (0.47–0.89)
	
*Vinorelbine*	
A	18.67 (6.58–30.76)
B	1.95 (0.84–3.06)
C	0.52 (0.09–0.95)
D	1.233 (0.80–1.66)
	
*Docetaxel*	
A	6.12 (2.53–9.71)
B	1.17 (0.73–1.61)
C	1.16 (0.80–1.51)
D	1.09 (0.73–1.44)

CI: confidence interval.

A: 24 h PEP005+drug.

B: 24 h PEP005 then 24 h drug.

C: 24 h drug then 24 h PEP005.

D: 24 h PEP005 then 24 h washout then 24 h drug.
